# Modeling the spatio-temporal dynamics of malaria parasite population genetics

**DOI:** 10.1186/1475-2875-13-S1-O17

**Published:** 2014-09-22

**Authors:** Edward Wenger, Joshua Proctor, Philip Eckhoff

**Affiliations:** 1 Institute for Disease Modeling, Bellevue, WA, USA

## 

A model of malaria transmission dynamics is presented that tracks the genetic barcodes of individual *Plasmodium falciparum* infections using seasonally-driven effective reproduction rates for clonal propagation, external importation, and the outcrossing of strains both within and between infections. We explore quantitatively the relationship between transmission intensity and genetic observations, for example the repeated observation of identical strains and their persistence across successive transmission seasons. Extending to a spatially connected network of human and parasite populations, we model the sensitivity of genetic sequencing to identify the relative contributions of local hotspots versus re-importation in sustaining transmission in pre-elimination settings. Finally, we model the effects of local transmission intensity and anti-malarial drug pressure on the population-level genetic signatures of emerging drug resistance.

